# Comprehensive Review of Alternative Proteins in Pet Food: Research Publications, Patents, and Product Trends in Plant, Aquatic, Insect, and Cell-Based Sources

**DOI:** 10.3390/foods14152640

**Published:** 2025-07-28

**Authors:** Phatthranit Klinmalai, Pitiya Kamonpatana, Arisara Thongpech, Janenutch Sodsai, Khwanchat Promhuad, Atcharawan Srisa, Yeyen Laorenza, Attawit Kovitvadhi, Sathita Areerat, Anusorn Seubsai, Shyam S. Sablani, Nathdanai Harnkarnsujarit

**Affiliations:** 1 Faculty of Agro-Industry, Chiang Mai University, Samut Sakhon 74000, Thailand; phatthranit.k@cmu.ac.th; 2 Department of Food Science and Technology, Faculty of Agro-Industry, Kasetsart University, Bangkok 10900, Thailand; fagipyk@ku.ac.th; 3 Department of Agro-Industrial Technology, Faculty of Agro-Industry, Kasetsart University, Bangkok 10900, Thailand; arisara.ari@ku.th; 4 Department of Packaging and Materials Technology, Faculty of Agro-Industry, Kasetsart University, Bangkok 10900, Thailand; janenutch.s@ku.ac.th (J.S.); khwanchatpromhuad@gmail.com (K.P.); atcharawan.s@ku.th (A.S.); yeyen.la@ku.th (Y.L.); 5 KU Vet Innova Nutricare Co. Ltd., Kasetsart University, Bangkok 10900, Thailand; attawitthai@gmail.com; 6 Department of Physiology, Faculty of Veterinary Medicine, Kasetsart University, Bangkok 10900, Thailand; sathitameen@gmail.com; 7 Department of Chemical Engineering, Faculty of Engineering, Kasetsart University, Bangkok 10900, Thailand; fengasn@ku.ac.th; 8 Department of Biological Systems Engineering, Washington State University, Pullman, WA 99164, USA; ssablani@wsu.edu

**Keywords:** pet food, alternative protein, sustainability, bioactive compounds, nutrition

## Abstract

The increasing demand for sustainable pet-food solutions has driven interest in alternative protein sources, as researchers seek to avoid allergenic foods while maintaining optimal pet nutrition. This review explores recent scientific publications, patent trends, and market trends relating to various alternative protein sources, including plant-based, aquatic, insect-derived, and cell-based sources. Their nutritional composition, functional properties, and potential benefits for pet health were assessed. Plant-based proteins, such as soy, pea, and lentils, provide essential amino acids and functional properties suitable for meat analogues. Microalgae and seaweed offer rich sources of omega-3 fatty acids, antioxidants, and bioactive compounds. Insect-based proteins such as black-soldier-fly larvae and mealworms are highly digestible and rich in essential nutrients, with additional benefits for gut health. Emerging cell-based proteins present a novel, lab-grown alternative with promising sustainability and nutritional advantages. While these protein sources offer significant benefits, challenges related to digestibility, palatability, regulatory approval, and consumer acceptance must be addressed. The emphasis of the present research is on current developments for industry uses and future potential. The analysis sheds light on the contributions of alternative protein sources to the promotion of sustainable and nutrient meals for pets.

## 1. Introduction

Over the years, there has been an increase in the growth of the pet-food industry, driven by increasing pet ownership and a heightened awareness of pet nutrition. The growing population of adult pets is a key trend, increasing the sales of high-quality pet protein foods. The market size of animal-based protein supplements has increased from $30.8 billion in 2024 to $33.6 billion in 2025, with a compound annual growth rate (CAGR) of 9.3% (The Business Research Company, Hyderabad, India, 2025). Common animal-based pet-food proteins are used in commercial pet food, such as chicken, turkey, beef, lamb, fish, etc. However, the animal-based ingredients used in the formulation of pet foods can cause an allergic reaction in a susceptible pet that can present as skin-related symptoms or as digestive and gastrointestinal symptoms [1,2]. Alternative proteins will be a key component of future food systems and a sustainable food source intended for both human and animal consumption. This shift aids in balancing the utilization of raw materials in response to global meat shortages caused by environmental concerns and plays a role in the mitigation of greenhouse gas emissions associated with global warming. Alternative protein sources with essential amino acids from plant-based sources, insects, microalgae, and cell-based or cultured meat have attracted interest as sustainable alternatives.

Pulse crops and pulse proteins are being used on a commercial scale as an alternative to animal protein-based food. The Food and Agriculture Organization (FAO) categorizes “pulse crops” as legumes primarily cultivated to produce dry seeds such as soybean, faba beans, chickpeas, beans, lentils, peas, and others [3]. However, many pet foods and treats still contain wheat and soy proteins, which are the most common food allergens in dogs and cats [4,5]. Seaweed and microalgae (e.g., Spirulina and Chlorella) are marketed as nutrient-dense food supplements rich in protein, essential fatty acids (e.g., omega-3 or omega-6), vitamins, minerals, and antioxidants [6]. Insect-derived proteins include those from black-soldier-fly larvae (*Hermetia illucens*), yellow mealworm larvae (*Tenebrio molitor*), and adult house crickets (*Acheta domesticus*), which are the main species used in animal feed [7]. Edible insects offer a source of high-quality digestible protein with essential amino acids that has been demonstrated to show support for canine digestion and has been used as novel protein source for dogs in order to prevent food allergic reactions [8,9]. Recently, Mota et al. (2024) [10] demonstrated that a blend of dietary macroalgae (*Ulva rigida* and *Fucus vesiculosus*) and the microalga (*Chlorella vulgaris*) has the potential for improving diet digestibility and fecal quality in dogs. Moreover, the production of algae and insects offers the prospect of a reduction in greenhouse gas emissions (GHG) and reduces water and land use requirements relative to other plant or animal-based alternatives [11,12]. Cell-cultured meat is also known as cultured meat, lab-grown meat, clean meat, synthetic meat, and animal cell-based meat; it is produced through the in vitro cultivation of animal cells [13]. Researchers and the food industry are increasingly interested in cell-based meat as an ethical and sustainable alternative to traditional animal-derived products. However, the use of cell-cultured meat as a raw material in functional or premium pet food remains limited.

The incorporation of alternative proteins into pet-food products presents both opportunities and challenges. While these proteins can enhance nutritional value and sustainability, factors such as palatability, digestibility, regulatory approval, and consumer acceptance must be carefully evaluated. This review aims to explore the potential of alternative protein sources in pet food, assessing their nutritional composition, functional properties, and overall impact on pet health. By examining the latest research and technological advancements, this study seeks to provide insights into the future of sustainable pet-food production.

## 2. Methodology

The exact Boolean search strings are provided in the figure captions of Section 3. All searches covered the period from 1 January 2014 to 31 December 2024. For Scopus, records were retrieved from the TITLE-ABS-KEY fields and limited to the document type “Article,”. Patent data were collected from World Intellectual Property Organization (WIPO) PATENTSCOPE, and filtered to obtain English-language abstracts and publication dates within the same timeframe. Product data were extracted from Mintel’s Global New Products Database (GNPD) by applying the same keywords in a full-text search and limiting the results to the “Pet Food” category.

## 3. Recent Trends in Publications, Product Launches, and Patents

### 3.1. Plant or Pulse Crop-Based Proteins

**Figure 1:**
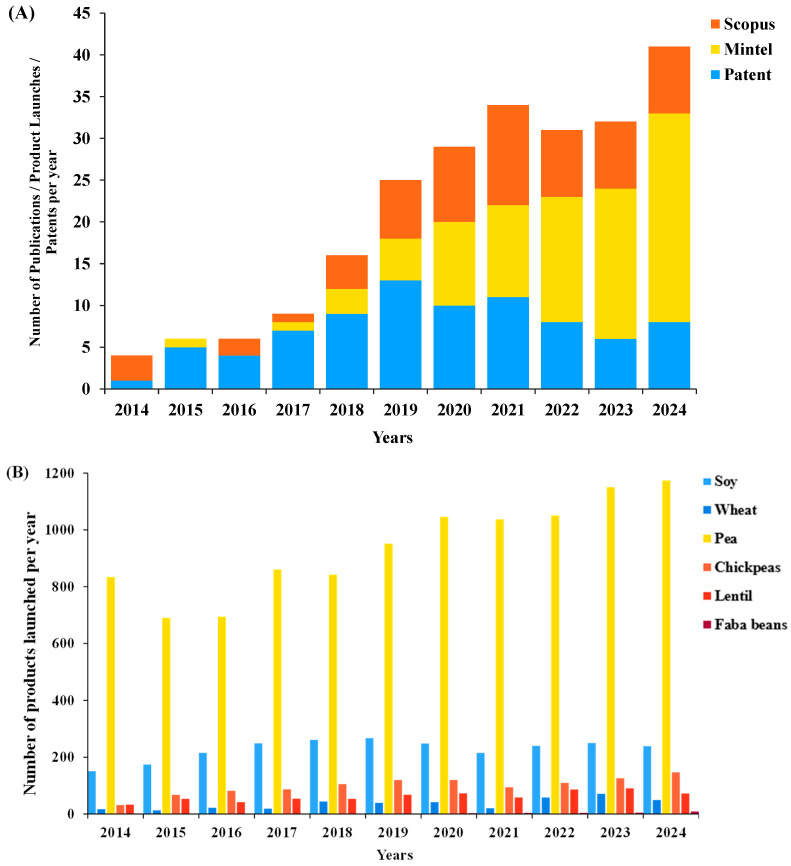
(**A**) Annual numbers of (i) publications (Scopus) using the following criteria: “Pet food”, “dog food” or “cat food” and the keywords “soy” or “pea” or “lentil” or “chickpeas” or “faba bean” or “pulse”; (ii) product launches listed in Mintel’s GNPD; and (iii) WIPO patents discovered using the following search terms: “soy proteins” or “wheat proteins” or “pea” or “lentils” or “chickpeas” or “faba bean” or “pulse” for and in January 2014 to December 2024. (**B**) Annual launches of pet-food products containing varieties of grains and claimed to be “plant based” on Mintel’s GNPD.

The demand for plant-based protein is mainly driven by soy and wheat proteins as substitutes for meat proteins [14]. Nowadays, scientific research levels and the numbers of patents worldwide relating to the use of wheat protein and legume proteins (such as lentils, soy, peas, chickpeas, and faba beans) as protein sources are showing slight increases (Figure 1A). In contrast, the number of new pet foods has undergone a significant increase each year from 2014 to 2024. However, soy and wheat are commonly found in various pet foods and have been shown to be common food allergens in dogs and cats [4]. Pea proteins are increasingly used as alternatives to soy proteins due to the low potential of pea proteins for allergic reactions [15], but pea proteins have poor gelling properties compared to soy proteins [16]. Foods subjected to scientific research published in the Scopus database and products marketed in Mintel’s GNPD increasingly utilize soy and pea protein in plant-based protein products. Cumulative totals from Mintel’s GNPD (2014–2024) indicate that soy-, wheat-, and pea-derived proteins, either alone or blended with other pulses, were the dominant plant-protein sources in complementary animal-based pet food (13,300 items) and in products explicitly marketed as plant-based pet food (89 items) (Figure 1B). Peas have been utilized as protein and fiber supplements in gluten-free, grain-free, and soy-free recipes with animal-based protein. These products serve as an alternative for junior and adult dogs with suspected food allergies. Lentils and faba beans have been classified as non-soy legumes that are gluten-free and that can serve as plant-based protein alternatives to meat [17,18]. Over the past decade, there has been a growing interest in the use of lentils and faba beans as alternative protein sources in pet food, as reflected in scientific research and new product launches. However, this trend has not widely become the subject of new patents (Figure 1A). According to Mintel’s global new product database (GNPD), lentils or faba beans are usually used as additives in recipes that blend pea or chickpea protein with animal-based protein, and which claim to offer both protein and a fiber-rich food. The products have been determined to support digestive health and help pets stay more energetic. The products launched with animal-based protein (703 items) are mostly for adult dogs (67.4%) and cats (32.6%). Only lentils are used in the pet foods claimed to be enriched solely with plant-based proteins (13 items).

### 3.2. Aquatic Plant Protein Sources

**Figure 2:**
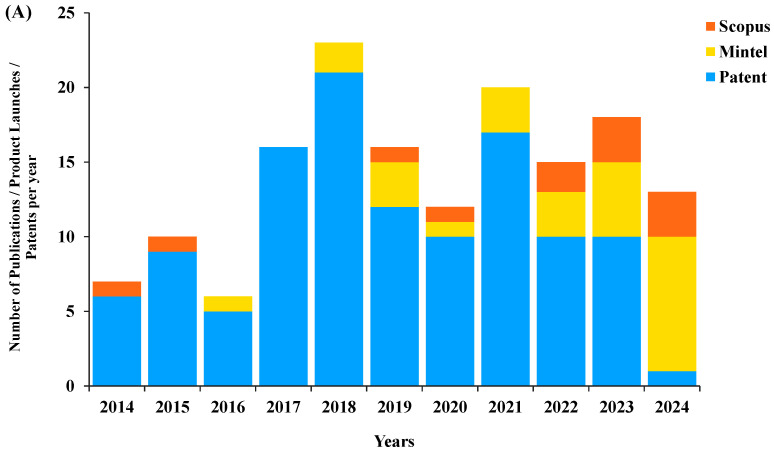

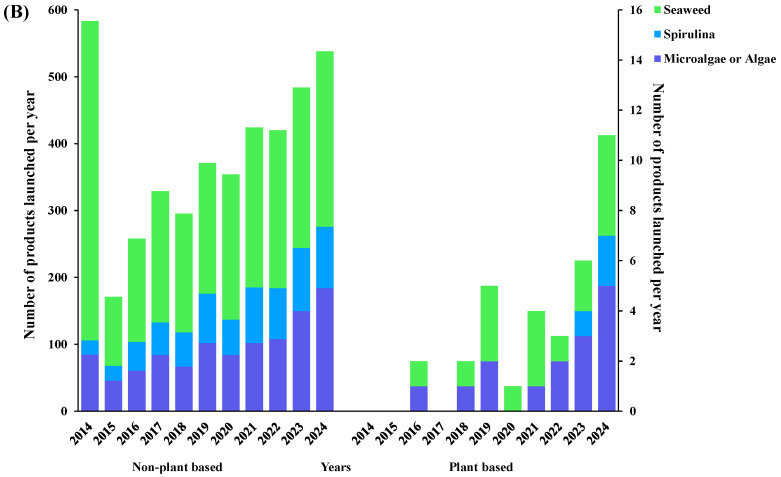
(**A**) Annual numbers for publications, pet-food products, and patents, using the following criteria: “Pet food”, “dog food” or “cat food” and the keywords “microalgae or algae” or “seaweed” or “spirulina” for Scopus; and claims “plant based” for Mintel’s GNPD (Global New Products Database) and World Intellectual Property Organization (WIPO) patents from January 2014 to December 2024. (**B**) Annual launches of pet-food products containing algae and seaweed, based on Mintel’s GNPD.

**Figure 3:**
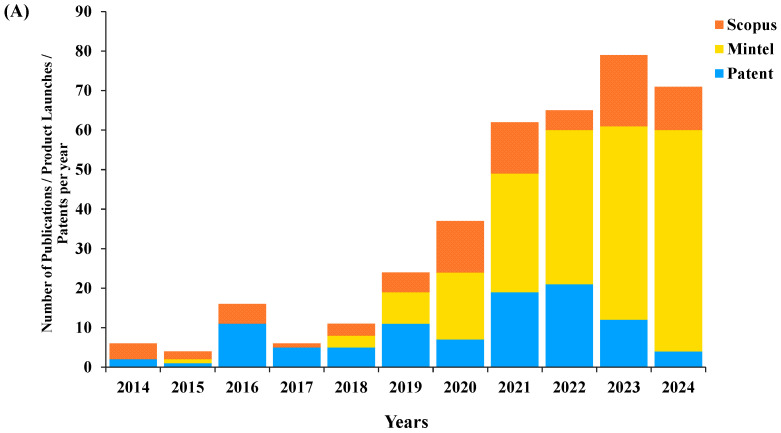

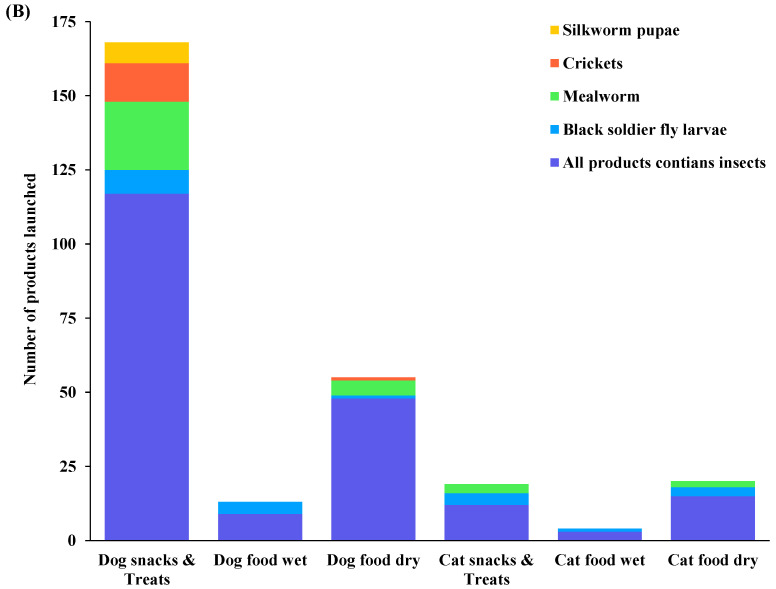
Numbers of relevant publications, pet-food products, and patents, using the following criteria: “Pet food”, “dog food” or “cat food” and the keywords “insect” or “mealworm” or “black-soldier-fly larvae” or “crickets”, for Scopus, Mintel’s GNPD (Global New Products Database), and World Intellectual Property Organization (WIPO) patents from January 2014 to December 2024.

Proteins derived from aquatic sources (including microalgae and algae) are rich in nutritional components such as proteins, amino acids, omega-3 and omega-6 fatty acids, pigments, vitamins, and antioxidants [19]. Proteins and pigments derived from microalgae are remarkably successful as nutritional supplements used for improving human and animal health. The utilization of microalgae in animal feed is a subject of interest, as evidenced by the scientific articles, globally launched products, and patent publications from January 2014 to December 2024 (Figure 2A). The number of patents related to microalgae used in pet food has risen above the number of published scientific articles, while new products are increasing annually. This suggests that microalgae-based product development is currently being driven more by commercial application than academic research, reflecting a shift toward proprietary innovation. Compared to plant-based protein sources, which show balanced trends across research, product launches, and patents (Figure 1A and Figure 3A), microalgae-based products are associated with a relatively higher level of patent activity, implying a faster transition into industrial use. The market for microalgae has encountered growth in the context of enhancing the nutrition, health, and wellness of pets. This has resulted in an increased interest in functional foods among pet owners and the pet-food industry. Mintel’s GNPD indicates that, as evident in in Figure 2B, the newly launched pet foods included those incorporating algae or seaweed in vegan and plant-based complementary formulas for adult dogs (26 items) and cats (1 item). The product-related claims only consist of the listing of plant-based protein sources as alternatives to animal protein and claims of suitability for dogs with food allergies and sensitive stomachs. However, the main ingredients are generally oats, rice, maize, soy, or pea, while algae or seaweed are mixed into the formula at levels of around 0.5% to 4% as a natural source of omega-3 fatty acids. The Pack in the US has blended dried algae, dried seaweed, and dried spirulina to create an oven-baked dried food for adult dogs. The product, launched in June 2024, is plant-based and vegan-friendly, and claims to support gut health in dogs. Spirulina algae powder is also incorporated into non-plant-based products due to its high contents of chlorophyll, minerals, and amino acids. It serves as a functional additive that supports intestinal health in dogs and the immune system in cats. Jin Kang Bao Pet Health Product in China has launched a dry food for adult dogs containing seaweed powder and spirulina powder, which is a blue-green algae with a very high protein content (up to 70%) and which contains all essential amino acids needed for muscle development and repair. The product claims to improve skin health and provide high-quality protein that aids in muscle gain.

### 3.3. Insect-Based Proteins

Insect farming has less substantial land and water requirements, a high feed conversion efficiency, and lower emissions of greenhouse gases (GHG) per kilogram of meat than traditional livestock (beef cattle and swine) [12]. Edible insects have superior protein content (50–80%) compared to other animal and plant proteins [20]. Furthermore, insects are high in nutrition, rich in essential amino acids, fatty acids, and vitamins and minerals, and comparable to pork, poultry, beef, chicken, fish, and soybeans [8,21]. The utilization of edible insects as alternative protein sources in functional pet foods for cats and dogs has attracted increasing attention from researchers and the pet-food industry (Figure 3A). Mintel’s GNPD indicates that black-soldier-fly larvae, mealworm larvae, crickets, and silkworm pupae *(Bombyx chrysalis)* have already been utilized as alternative protein sources in functional dog foods (85.3%) and cat foods (14.7%) from 2014 to 2024 (Figure 3B). Black-soldier-fly larvae constitute 42.2% of the insects utilized for pet food, and are the most used, followed by mealworms at 37.3%, crickets at 13.7%, and silkworm pupae at 6.86%. The protein derived from black-soldier-fly larvae (*Hermetia illucens*) has been utilized in snacks and treats for dogs (69.8%) and cats (30.2%) over the past decade, as reported by Mintel’s GNPD. A mono-protein insect snack made from black soldier fly (25–100%) as a complementary product in dog treats has been marketed as a product intended for dogs aged from six months to adult. The hypoallergenic product is ideal for sensitive dogs and demonstrates greater digestibility. The recipes for many premium chews, kibbles, and dry cat foods blend black-soldier-fly protein with animal-derived ingredients such as lamb, chicken, salmon, and turkey. These items are claimed to be suitable for cats of all life-stages with sensitive digestion, improving immunity, promoting metabolism, and minimizing the risks of food allergies. A functional dog food containing mealworm proteins as a high-protein domestically sourced powder claims to be suitable for sensitive and hypoallergenic dogs and suited for all life-stages. Most snacks, treats, and dry foods for dogs (81.8%), cats (15.2%), and both dogs and cats (3.03%) are formulated with a single protein source of mealworm combined with vegetable blends, and free from meat, grains, or gluten. These products claim to be associated with improved digestion, the boosting of immunity, and the reduction of inflammation. Insects possess a unique chitinous exoskeleton that, while indigestible in large quantities, can act as a prebiotic fiber in smaller amounts [22]. This chitin can promote the growth of beneficial gut bacteria, potentially leading to improved digestive health. Although edible insects can be a valuable protein source, their inclusion may lead to increased amounts of indigestible carbohydrates, potentially causing digestive issues and gastric discomfort in dogs [9,23]. Only a few treats containing an insect protein mix are now available. Rolf C. Hagen Inc., of Germany, launched a cat treat which contains 96% sustainable protein derived from a black-soldier-fly larvae and mealworm mix. Matina GmbH, Germany, launched highly premium adult dog snacks with dried mealworms (*Tenebrio molitor*) and dried crickets (*Gryllus testaceus*) as protein sources. Pup Pup Foods Ltd., Ireland, launched dental chews for dogs that blend black-soldier-fly larvae and crickets as nutritious protein sources for the promotion of healthy teeth and gums. Dog snacks and treats can contain cricket or silkworm pupae as a high-protein additive, but these are not utilized in complementary cat foods. Cricket protein is claimed to be more easily digestible than meat and is rich in amino acids, vitamin B_12_, iron, and fiber, and is suitable for all ages, while silkworm pupae protein is claimed to be rich in amino acids and omega-3, omega-6, and omega-9, and is suitable for dogs from four months of age.

## 4. Alternative Protein Sources in Pet Food

### 4.1. Plant or Crop-Based Proteins

The environmental footprint of pet food, particularly foods for dogs and cats, is substantial. In the United States, the meat consumed by these pets is estimated to generate as much as 80 million tons of methane and nitrous oxide. Sustainability, broadly defined, means meeting present needs without compromising future generations’ ability to meet their own needs. One proposed way to make pet food more sustainable is to replace animal-based protein with plant-based protein, thereby reducing resource consumption and the associated carbon footprint [24,25]. Plant proteins offer a range of useful functional properties, including solubility, viscosity, water and oil retention, foaming, emulsification, and gelation. These properties make them suitable for creating meat analogues that mimic the texture, appearance, flavor, and mouthfeel of meat products. While nearly any plant protein could be used for this purpose, soy and pea proteins (among legumes) and wheat gluten (among cereals) are the most common choices due to their widespread availability, affordability, and ease of processing [26,27]. Meat analogues based on plant proteins are being developed for use not only in the food sector, but also in the pet-food sector (Table 1). Wehrmaker et al. (2021) [28] produced a Couette-cell meat analogue (CCMA)-based pet food from soy protein isolates and wheat gluten, exploring different canning media: water, jelly, and gravy. Sterilization increased moisture content most significantly in the water-canned CCMA (82 g uptake), followed by jelly (57 g) and gravy (48 g). This increased moisture led to decreased hardness and springiness. Nitrogen loss during sterilization was highest in the jelly-canned CCMA (1 g), while fat content remained stable across all media. During storage, both composition and texture remained consistent. Overall, standard pet-food sterilization significantly altered the texture of the CCMA chunks, suggesting that fresh CCMA may have different properties than processed meat chunks. In another study, Wehrmaker et al. (2022) [29] successfully produced chunk-shaped pet food from wheat gluten and soy protein isolate (MaSoy) and wheat gluten and pea protein isolate (MaPea), using a shearing method at 120 °C for 30 min. They analyzed the changes in amino acid content after shearing and sterilization (26 min at 125.5 °C and 1.36 bar). After processing, the amounts of nitrogen-based amino acids decreased by an average of 31.0 ± 1.86% for MaSoy and 14.4 ± 2.76% for MaPea. However, compared to animal-based pet food, this plant-protein pet food remained more balanced or less affected, even after extensive thermal processing. Wehrmaker et al. (2024) [30] developed a wheat gluten-based pet food with a mix of three different proteins: MaSoy, MaPea, and faba bean concentrate (MaFaba). Crude protein contents ranged from 72.8% to 87.8%, while the crude fat content was generally low, with MaPea in gravy exhibiting the highest levels. Importantly, their research demonstrates that these plant-based meat analogues provide excellent digestibility, ensuring ample amino acid and phosphorus availability for optimal pet health. However, essential amino acids like methionine and taurine are commonly deficient in plant-based proteins.

### 4.2. Aquatic Plant Protein Sources

Marine-based plants such as microalgae, macroalgae, and spirulina are starting to be commonly used in pet food due to their composition, which is rich in nutrients, protein, polyunsaturated fatty acids, minerals, and polyphenols. These nutrients are commonly used as anti-inflammatory, prebiotic, antioxidant, or immunomodulatory agents. Microalgae are unicellular photosynthetic microorganisms that have been suggested for use in sustainable food production due to their ability to convert inorganic and organic compounds into biomass with higher nutrient contents than terrestrial plants (Table 2). Microalgae-based pet food is commonly provided as a supplement to support pet health. Algal-based diet supplements contain high amounts of polyunsaturated fatty acids (PUFAs). *Nannochloropsis oceanica* has been reported to promote eicosapentaenoic acid (EPA; C20:5 n-3) content in a supplemented diet [31]. The algal diet also contains high and varying amounts of essential amino acids, with arginine, lysine, and leucine being the dominant amino acids found in *C. vulgaris* blends with some macroalgae [10]. Cabrita et al. (2023) [31] and Delmonte et al. (2023) [32] developed dietary supplements for dogs using the microalgae species *Chlorella vulgaris, Nannochloropsis oceanica,* and *Tetradesmus obliquus*. The genera Turicibacter and Peptococcus were abundant, and moreover, it was observed that the number of defecations decreased, which correlated with a healthier gut. Similarly, Mota et al. (2024) [10] found that the *C. vulgaris* diet was high in healthy genera such as Turicibacter and Blautia, which were the most abundant in all samples, while also promoting metabolized energy and improving fecal quality. Delmonte et al. (2023) [32] used the microalgae species *Chlorella vulgaris, Arthrospira platensis, Haematococcus pluvialis*, and *Phaeodactylum tricornutum* as potential iron sources for dogs. They found that *C. vulgaris* had the highest iron content and iron bioaccessibility. Spirulina (*Arthrospira platensis*) is a cyanobacterium belonging to the phylum Cyanobacteria. Its photosynthetic nature makes spirulina an evolutionary bridge between green plants and bacteria. It is commonly used as a dietary supplement that provides health benefits due to its contents of protein, PUFAs, fiber, and pigments (chlorophyll, β-carotene, and C-phycocyanin) [33]. A diet including supplementary spirulina tablets was accepted by both dogs and cats, whether the tablets were given alone or mixed with complete meals, and it did not have any effect on fecal score, vomiting, scratching, or behavioral status. Furthermore, dogs supplemented with spirulina demonstrated a higher immune status with a greater vaccine response and higher levels of fecal immunoglobulin A (IgA) [34]. Macroalgae, mostly seaweed such as *Kappaphycus alvarezi, Gracilaria* spp., *Gelidiella* spp., *Hypnea* spp., and *Eucheuma* spp., have been reported to have beneficial effects on gut health and immunity in animals [35]. Srinivas et al. (2024) [35] found that adult dogs supplemented with *Kappaphycus alvarezii* and *Gracilaria salicornia* had higher levels of Bifidobacteria, while Clostridia levels were lower in fecal concentrations, indicating that a seaweed-based diet could promote gut health and the ability to digest dietary fiber. The study of antioxidants in the dogs’ blood showed that seaweed supplementation resulted in a higher glutathione concentration (GSH) reduction and lower lipid peroxidation. *Ascophyllum nodosum* (AN), or kelp, is a brown algae that contains glycans and secondary metabolites like phlorotannins, which have potential anti-inflammatory and prebiotic actions [36,37]. This type of algae has been used as a dietary supplement for pets. The combination of AN and hydrolysate protein increased the abundance of beneficial bacteria, namely *Ruminococcaceae* and *Rikenellaceae*, in fecal concentrations. After adding *Bacillus subtilis* to the AN + hydrolysate protein mixture, an association with greater numbers of *Enterococcus* and *Bacillus* was observed, indicating the beneficial effects of probiotic supplementation. Fermented seaweed, *Kappaphycus alvarezii*, could help maintain the quality of cats’ skin and hair coats [38]. A supplement for Ragdoll kittens, based on brown algae (*Laminaria* sp.) treated using an enzymolysis process and including the probiotic *Saccharomyces boulardii*, showed higher abundance of *Bacteroidetes*, *Lachnospiraceae, Prevotellaceae,* and *Faecalibacterium*, which are beneficial bacteria found in a healthy gut microbiome. The observed palatability, digestibility, gut microbiome stability, immune system benefits, and other health benefits derived from marine plant-based diets for cats and dogs suggest that microalgae and macroalgae are potential sources of the main ingredients used in supplementation (Table 2). Further investigations into processability, supplement form and size, dosages for specific purposes, and specific cat or dog species could be potential approaches for future research.

**Table 2:** Aquatic plant-based proteins in pet foods.

Protein Source	Food Process	Product	Factors of Investigation	Method of Investigation	Major Finding	References
Macroalgae (*Ulva rigida, Fucus vesiculosus*), Microalga (*Chlorella vulgaris*)	Spray-dried mixed algae were added to commercial extruded dog food for medium-sized adult animals	Algal diet mixed into kibble for dogs	Diets: 0% (A0), 0.5% (A0.5), 1% (A1), and 1.5% (A1.5) algal blends	• Palatability • Digestibility • Chemical composition (ash, protein, crude protein, neutral detergent fiber, acid detergent fiber, amino acid, fatty acid) • Fecal microbiota	• Algal blend diet: high minerals, n-6 and n-3 PUFAs • Algae did not affect initial taste, but dogs preferred the control diet • A 1.5% algal diet improved digestibility without affecting intake • Algal blends A1.5 and A1 reduced fecal output and production • High digestibility: lower input, higher energy, reduced feces • Algal blend: *Ligilactobacillus*-specific fecal microbiota effect	[10]
Spirulina (*Arthrospira Platensis*)		Tablet for for cats and dogs	Spirulina dosages: 0.4 g (cats/small dogs), 0.8 g (medium dogs), 1.2 g (large dogs)	• Palatability • Spirulina’s effect on pet gastrointestinal health markers • Nutritional profile	• High acceptance of spirulina tablets was observed in cats and dogs, both alone and mixed with food • Gastrointestinal tolerance of spirulina tablets was high in cats and dogs, with rare vomiting and diarrhea • No behavioral changes were observed in dogs fed spirulina diets	[33]
Microalgae (*Chlorella vulgaris, Nannochloropis oceanica, and Tetradesmus obliquus*)		Powder for dogs	Algae were added to the reference diet at 0.5–1.5%	• Palatability • Digestibility • Proximate analysis	• Dogs exhibited a preference for the reference diet compared to the 1.5% microalgae diet • Dietary composition, intake, and fecal output remained largely unaffected by microalgae supplementation (≤1.5%) • *C. vulgaris* enhanced protein digestibility • Microalgae diets boosted beneficial gut microbiota, enhancing gut health and immunity.	[31]
Microalgae *(Tetradesmusobliquus*, *Chlorella vulgaris,* and *Nannochloropsis oceanic)*	Spray dryer	Powder for dogs	-	• Amino acid • Metabolomic profiling (LC-MS/MS) • Proximate analysis	• Essential amino acids met dogs’ requirements, except methionine/cysteine • Microalgae *N. oceanica, T. obliquus*, and *C. vulgaris* lack sufficient linoleic, linolenic, arachidonic, and eicosapentaenoic acids for dog food requirements. • Species differentiation was driven by glycolipids, glycerolipids, and phospholipids in untargeted metabolomics	[39]
Algae *(Ascophyllum Nodosum)*	Prepared by manufacturer	Powder for dogs	• Brown algae A.N. ProDen™ (active) • Microcrystalline cellulose powder (placebo)	Metabolomic profiling data (100–1100 Da)	• Positive clinical effects and the elimination of salivary metabolites were observed in dogs after 30 days of *A. nodosum* supplementation	[37]
Microalgae species (*C. vulgaris*, *A. platensis*, *H. pluvialis*, and *P. tricornutum*)	Prepared by Micoperi Blue Growth (Ravenna, Italy)	Algal pet supplements		• AAS Iron Analysis • G-75 SEC Iron Speciation Analysis	• *C. vulgaris* exhibited significantly higher iron content (1347 ± 93 μg/g) compared to *H. pluvialis* (216 ± 59 μg/g) • Bioaccessibility: 30% (*C. vulgaris*), 31% (*A. platensis*), 30% (*H. pluvialis*) • *C. vulgaris*: high iron, good bioaccessibility	[32]
Seaweed *(Kappaphycus alvarezii*, *Gracilaria salicornia)*		Powder for dogs	Basal diet with: Control; *K. alvarezii* (1.5%, 3%); *G. salicornia* (1.5%, 3%)	• Feed intake • Palatability • Digestion • Immune response • Blood antioxidant and peroxidation analysis	• Seaweed supplementation: no significant impact on digestibility, intake, or weight • *Gracilaria salicornia* supplementation, compared to *Kappaphycus alvarezii*, significantly improved gut health, antioxidants, and immune response, with no adverse effects on palatability or nutrient utilization	[35]
Brown seaweed *(Ascophyllum nodosum) with and without B. subtilis*	-	Powder for dogs	Control, HP, HP+Algae (HPA), HP+Algae+*B. subtilis* (HPAB)	• Chemical analysis • Clinical Analysis • Gut microbiota • Fecal metabolite analysis	• Increased Ruminococcaceae/Rikenellaceae in HPA-supplemented dogs, with *Bacillus* abundance unique to HPAB • Higher acetate in HPA-fed dogs • HPA had higher isovalerate and isobutyrate levels • HPAB diet increased fecal butyrate	[36]
Brown algae *(Ascophyllum nodosum, Undaria pinnatifida,* and *saccharina japonica)* Red algae *(Palmaria palmata)*	Freeze dried	Powder for adult dogs	Control diet: poultry protein, grains, fats, fish meal, linseed oil, eggs, minerals; with 15 g/kg algal powder supplement	• Fecal bacterial analysis • Lyophilized fecal IgA by commercial dog ELISA	• Algal supplementation had no effect on fecal chemistry, IgA, or nutrient ATTD • No microbiological effects were observed from algal ingestion • Algal supplementation (15 g/kg) ineffective relative to fecal parameters.	[40]
Brown algae *(Ascophyllum nodosum L)* (AN)		Powder for dogs	Control diet (CTR) and CTR supplemented with 0.3% and 1% *A. nodosum* powder	• Palatability • Chemical Composition • Metabolic Analysis	• Food consumption varied between CTR and CTR + high AN groups • Palatability tests showed varying first-choice behavior • *A. nodosum* dose-dependently inhibited dry matter intake	[41]
*Spirulina* *(Arthrospira platensis)*	Spray dryer	Powder for dogs	Control diet (29% protein, 36% carbohydrate, 19% fat, 1.4% fiber) and Control + 0.2% spirulina	• Plasma RFFIT antibodies • Fecal antibody ELISA, canine CRP, and PCR-TTGE for gut microbiota analysis	• Spirulina improved canine immune status: increased rabies vaccine response and fecal IgA • Spirulina regulates canine mucosal and systemic immunity • Spirulina improves dog gut microbiota stability	[34]
Seaweed *(Kappaphycus alvarezii)*	Fermentation dried seaweed	Powder in capsules for cats	Control diet, Control + 2% fermented seaweed	• Post-feeding physiological and digestive health evaluation	• Highly digestible (fiber, fat, protein) • Fecal lactobacilli slightly increased at 2 months • No significant effects observed from supplementation on behavior, feed intake, weight gain, or fecal quality • Improved cat skin quality (3.89 to 4.2)	[38]
Seaweed (*Laminaria* spp.) combined with *Saccharomyces boulardii*	Enzymolysis	Powder for Ragdoll kittens	Control, Seaweed-Enzymolysis (SE), and *S. boulardii* (SB)	• Plasma • Microbiota • SCFA Analysis	• Enzymolysis seaweed improved Ragdoll kitten immunity • SE group: reduced kitten intestinal permeability and inflammation • SE group: higher levels of Bacteroidete, Lachnospiraceae, Prevotellaceae, Faecalibacterium • No SCFAs effect from enzymolyzed seaweed	[42]

### 4.3. Insect Alternative Protein in Pet Foods

In the present era, the pet-food industry is looking for new alternative protein sources, and insect protein has gained attention due to its fast production times, high nutritional value, and small cultivation area (Table 3). Insects, especially black-soldier-fly larvae (*Hermetia illucens*), mealworms, crickets, and silkworm pupae, are potential raw materials with many benefits for pets [43,44]. Recent studies have shown the efficacy of insect protein in industrial feed processing, such as dry extrusion, semi-wet cooking, and hydrolyzed formulations. In addition to industrial production efficiency, insect protein also has a positive effects on digestibility, the digestive system, and the health balance of pets [45,46]. The digestibility-related and nutritional benefits of insect protein are significant. The digestibility of insect protein affects the properties of the digestive system and excretory system, with several studies comparing insect protein with general animal proteins in terms of essential nutrients. The use of foods compatible with the intestinal properties of pets can result in higher levels of protein digestion, with some black-soldier-fly larvae-based protein showing levels of digestibility higher than those of poultry-based foods. In extruded pet foods, the industry has created a driving force working to support the nutritional properties of the gut properties of dogs [44]. In not showing a significant effect on the properties of gut bacteria, pet foods such as crickets and silkworms, on the contrary, create a balanced intestinal ecosystem in pets, which will have a long-term positive effect on the health of pets [45]. The effects of insect protein on gut microbiota and feces quality reflect the composition of the gut microbiota balance; studies on insect-based diets have shown changes in the gut microbiota of dogs fed insect-based diets that have been associated with higher levels of beneficial bacteria, such as Bifidobacterium, which affects the quality of dog feces and reflects the balance of the gut microbiota [43]. Insect-based diets also reduce the amounts of bacteria that are harmful to the canine’s gut, such as Turicibacter, and increase the amount of beneficial bacteria, which results in better gut health and immune response [45]. In addition, the process of hydrolyzing black-soldier-fly protein promotes the growth of beneficial bacteria and helps to enhance the characteristics of good cells to support the immunity and health of pets [47]. In addition, the addition of cereals mixed with insects, such as fermented oats and BSFL-based diets, can reduce cholesterol levels, inhibit the inflammatory cytokines (TNF-α, IL-10) that may lead to cardiovascular problems in older dogs, and better control the level of health hazards to animals [48]. The industry tends to focus on the use of alternative proteins. Consumers are interested in the use of alternative proteins from insects but still pay more attention to the appearance of the pet food [49]. Insect-based treats, pellets, or kibbles are generally more acceptable to consumers than are whole insects, due to reduced visual aversion and better palatability among pets [43]. Furthermore, a growing awareness of the sustainability-related, hypoallergenic, and digestive health benefits has been shown to increase the willingness of consumers to accept insect-based products. Therefore, the shift to feeding pets alternative proteins made from insects is an important and interesting aspect of the pet-food industry in the context of alternative proteins.

**Table 3:** Insect-based proteins in pet foods.

Protein Source	Food Process	Product	Factors of Investigation	Method of Investigation	Major Finding	Ref
Fish meal (FM), Meat and bone meal (MBM), Corn gluten meal (CGM), Soybean meal (SBM), Mealworm meal (MM), Yeast extract (YE)	-	Dry dog food with 30% ingredient replacement	Digestibility, energy, and fecal analysis	Beagle feeding trial: digestibility, energy, and fecal assessment	• Digestibility and fecal moisture varied significantly across diets • Fish and corn gluten meal showed highest digestibility • Meat and bone meal showed lowest energy values • Soybean meal showed highest fecal moisture • Mealworm meal had high energy but lower digestibility	[44]
Black-soldier-fly larvae	Dry extruded	Dry extruded foods for cats	Pet-food digestibility, fecal and microbial analysis, fermentation, and blood profiles	Digestibility (fecal analysis), fermentation, and comprehensive blood analysis	• High palatability • High digestibility, relative to control: poultry meal • Microbiota: lower diversity, higher Bifidobacterium • No clinically relevant differences observed	[43]
House cricket and Mulberry silkworm pupae	Dry	Semi-moist	Fecal microbial diversity and composition	A 29-day feeding trial, including fecal sample, 16S rRNA analysis	• No significant alpha diversity differences (Chao1, Shannon, Simpson) between groups • Increased *Lactobacillus* and *Corynebacterium genera* • Decreased Turicibacter in AD and BMp groups	[45]
Black-soldier-fly larvae protein and fat	Extrusion	Dry foods for dogs	Digestibility, microbial fermentation, and metabolic markers	Fecal (digestibility, SCFAs, BCFAs, microbiota) and serum (biochemistry, antioxidant, inflammatory) analysis	• Defatted BSFL shows decreased digestibility • No digestive issues from black-soldier-fly larvae fat • Defatted BSFL protein alters fecal pH, SCFA/BCFA, and gut microbiota • Black-soldier-fly larvae fat group major *Terrisporobacter* and *Ralstonia/* metabolome no changes	[49]
Black-soldier-fly larvae meal and Fermented oats	Pelletized, dried at 70 °C, then frozen (−20 °C) for storage	Pelleted food for senior dogs	Nutritional intake, digestive function, skin integrity, and immune parameters	Fecal scoring, blood parameters (CBC, biochemical, cytokines, IgG), and skin hydration/lipid profiles	• Black-soldier-fly larvae diet showed cholesterol reduction, with no other significant adverse effects	[48]
Black-soldier-fly larvae meal	Extrusion (107 °C, 30 bar, 1 min), then dried (120 °C, 30 min)	Dry extruded food for dogs	Nutrient digestibility and fecal quality assessment	Beagle fecal digestibility: poultry and BSFL meal (moisture, consistency, DM)	• Enhanced protein and fat digestibility, and lower fecal dry matter, compared to poultry meal	[50]
Black-soldier-fly larvae meal		Dry extruded food for dogs	Nutrient digestibility (Crude protein, Fat, Fiber, Organic matter, Calcium, Phosphorus)	Digestibility trial: 6 dogs, TFC/marker/enzymatic methods, in vivo/in vitro correlation	• Enhanced calcium digestibility; fat, organic matter, and phosphorus digestibility comparable to traditional diets • Reduced crude fiber digestibility due to chitin, with valid in vitro and in vivo digestibility assessments	[46]
Black-soldier-fly larvae (BSFL)	Extrusion	Kibble at 30% dry matter (DM)	Digestibility and fecal parameters	Beagle diet study: BSFL vs. PM, digestibility and fecal analysis	• BSFL meal is a highly digestible, sustainable alternative to poultry meal for dogs	[50]
Black-soldier-fly (BSFL) protein derivatives	-	Pet-food formulation (protein meal, hydrolysates)	Antioxidant and immunomodulatory properties	BSFL protein evaluation: nutritional, antioxidant, and immunomodulatory properties	• BSFL protein derivatives for enhanced antioxidant and anti-inflammatory pet-food formulations.	[47]
Mushroom and Mealworm	-	Grounded dog-food attractant	Enzymatic hydrolysis and palatability characterization	Hydrolysis optimization, aroma analysis, and canine palatability testing	• Aroma compounds significantly improved the lower palatability of mushroom and mealworm attractants	[51]
Black-soldier-fly larvae (BSFL), Housefly larvae (HF), Yellow mealworm (YMW)	Cryopreserved (−20 °C) and 1.25 mm sieved larvae	Freeze-dried insect-based protein for dog food	Digestibility, microbial fermentability, and metabolite analysis	A 48 h dog fecal fermentation; gas production and 1H NMR metabolite analysis	• Hydrolyzed Feather (HF) and Yeast Mannan Wall (YMW) exhibited superior nitrogen and amino acid digestibility compared to Black-soldier-fly larvae (BSFL) • YMW showed higher fermentation	[52]

### 4.4. Cell-Based Sources

Cultivated meat (also known as cell-based meat, cultured meat, in vitro meat, lab-grown meat, or synthetic meat) is an alternative protein source developed to address the sustainability, public health, and animal welfare concerns associated with conventional meat production. As animal agriculture is a leading contributor to climate change, cultivated meat offers a potential solution. Furthermore, cultivated meat could be used in pet food, helping to resolve the meat supply issues faced by the growing pet-food market in recent years [53]. Cultivated chicken meat is currently available for human consumption in Singapore and Israel, and numerous startups are developing additional cultivated meat products for various markets [54]. The U.S. Food and Drug Administration (FDA) has approved cultivated chicken products from both Upside Foods and Good Meat (a subsidiary of Eat Just) for sale in the U.S. market. Similarly, Singapore has approved Eat Just’s cultivated chicken for the Singapore market, and Israel recently approved Aleph Farms’ kosher cultivated beef steaks for sale in Israel [55]. The advantages of using cultivated meat as a pet protein source include the reduced environmental impact of pet diets, decreased farm-animal suffering, and additional benefits. Moreover, its use can reduce the reliance on plant-based diets for pets [56]. Specifically, cultivated meat-based pet food would significantly reduce the risks of spreading food safety pathogens, zoonotic diseases, and antibiotic-resistant bacteria [53]. The antibiotic-free manufacturing process and the aseptic conditions required for cell growth in bioreactors contribute to these public health advantages. However, cultivated meat has not yet been produced at scale for either human or pet consumption. Several technical challenges must be overcome to make cultivated meat-based pet food at prices accessible to consumers [53]. Additionally, since it is a novel ingredient, there is no existing evidence on the long-term effects of feeding cultivated meat to dogs and cats. While, in principle, cultivated meat can be safe for long term consumption and nutritionally adequate, and potentially even superior to conventional meat due to the possibility of nutritional enhancement, manufacturers must demonstrate the safety and nutritional soundness of cultivated meat-based products to gain regulatory approval and encourage consumer adoption. The collaborative efforts of veterinarians, food scientists, and technicians will be critical for the development of this new ingredient for sustainable pet food, a process involving product development, safety and nutrition assessments, research, and consumer education [54]. In the future, cultivated meat offers a potential tasty, low-carbon, and healthy protein source, one with the potential to eliminate the use of farmed animals from the pet-food industry.

## 5. Patents Relating to Alternative Proteins for Pet Foods

The pet-food industry has experienced a growing wave of innovations centered on alternative protein sources, driven by concerns for sustainability and health. Patents related to alternative proteins for pet foods are shown in Table 4. USPET Nutrition et al. (2020) [57] combined animal-based proteins (such as fish or meat flakes) with plant-based proteins (like textured soybean protein) to achieve a balanced formulation that met specific nutritional needs, including maintaining methionine levels between 0.62% and 1.5% on a dry weight basis. The textured plant protein, such as fibrous soybean protein, is processed into imitation meat chunks that closely mimic the appearance and texture of real meat. The final product consists of meat flakes, imitation meat chunks, and additional ingredients like vegetables, grains, and oils. The manufacturing process involves mixing, extrusion, heating, and cooling to develop a structured, fibrous texture in the plant-based protein components. Unicharm Corporation (2019) [58] incorporated pea protein and egg-based ingredients (including dried egg products and egg whites) to produce meat-like chunks. Alternative proteins are blended with meat components to create a binding matrix that improves texture and structural stability. The processing involves scraped surface heat exchangers, which handle the meat slurry and alternative proteins under precise conditions. The final product replicates the fibrous texture, striations, and visual characteristics of real meat chunks, making it suitable for both dry and wet pet-food formulations. Kyu-Taek Hwang (2023) [59] outlines a pet-food formulation incorporating Achyranthes japonica Nakai extract alongside a mix of conventional and alternative protein sources, including grains, meat, fish, eggs, and seaweed. It describes a method for encapsulating or blending the extract to optimize its integration. By combining traditional and alternative proteins, the formulation enhances the pet food’s nutritional value. The addition of *Achyranthes japonica* Nakai extract aims to deliver antioxidant benefits while ensuring a balanced supply of essential amino acids, vitamins, and minerals to meet pets’ dietary requirements. Qingdao Yalute Food Co., Ltd. (2022) [60] has described a high-protein pet snack formulated with alternative proteins, including plant-based artificial meat made from king oyster mushroom powder, plant protein powder, and textured soy protein. This protein blend offers essential amino acids comparable to those in animal proteins, ensuring pets receive adequate nutrition. Additionally, the product is low in cholesterol and saturated fatty acids, helping to reduce the risk of obesity and other diet-related health issues in pets. Shanghai Liangrun Industrial Co., Ltd. (2024) [61] has combined plant-based proteins, including pea protein and fermented soybean, with traditional animal-derived proteins such as chicken breast, beef, salmon, and seabass. These alternative proteins help maintain a high overall protein content (≥55%), providing essential nutrition while promoting sustainability and functionality. The formula incorporates multi-layer microencapsulation, embedding alternative proteins and functional ingredients within carbohydrate matrices to enable controlled nutrient release and gradual absorption. Clinical trials demonstrated notable reductions of 15% in both blood glucose and triglyceride in pets consuming the food. Halldorsdottir et al. (2015) [62] show a method for producing high-quality aquatic protein hydrolysates (APHs) through enzymatic hydrolysis of aquatic protein sources, such as fish, aquatic mammals, crustaceans, and mollusks, combined with natural antioxidants from marine algae like *Fucus vesiculosus* extract. The inclusion of marine algae extracts during hydrolysis helps prevent oxidation and improves the functional properties of the hydrolysates. Sensory evaluations revealed a significant reduction in bitterness and off-flavors. With high bioavailability and digestibility, these APHs serve as a valuable ingredient in pet-food formulations, offering anti-hypertensive and immune-boosting benefits. Air Protein Inc., (2022) [63] has explored the use of microbial protein sources in high-protein food formulations, including pet food. It emphasizes single-cell proteins derived from microorganisms like *Cupriavidus necator*, which can be processed into meat analogues or integrated into pet-food products. These proteins can be customized to supply essential amino acids, ensuring a nutritionally complete and balanced diet for pets. Inventor (2024) [64] has utilized alternative proteins to create nutritionally balanced, sustainable, and easily digestible formulations. The composition includes pea protein, soy protein, wheat gluten, chicken, beef, fish protein, and meat by-products, all selected to provide complete amino acid profiles for optimal pet nutrition. Shanghai Peitong Technology Co., Ltd. (2023) [65] has detailed a pet-food formula made from fresh meat, and enriched with yeast probiotics and seaweed powder. The seaweed powder supplies vital minerals like iodine, which aids thyroid function, and contains antioxidants and bioactive compounds. Daesang Farmsco Co., Ltd., (2008) [66] has outlined a dog food formula that uses *Chlorella* as an alternative protein source. Experimental trials demonstrated multiple benefits, including improved coat shine and overall health. The study also found that *Chlorella*-based food reduced fecal moisture content, promoting better fecal consistency and reductions in odor. Additionally, dogs showed a preference for the *Chlorella*-enriched diet over traditional feed. Mars, Incorporated (2021) [67] has focused on the use of microalgae biomass as an alternative protein and functional component in pet-food products. Microalgae biomass (such as *Porphyridium cruentum*, *Chlorella*, *Haematococcus*, *Scenedesmus*, and *Euglena*) acts as a heat-settable binder in restructured meat analogues for wet pet food. This method offers a cost-efficient and environmentally friendly solution for protein substitution. The Netherlands Patent Office (2021) has investigated [68] the use of insect protein hydrolysates as an alternative protein source in pet food. Insect protein hydrolysates offer highly digestible, water-soluble proteins rich in essential amino acids, peptides, and bioactive compounds. Additionally, bioactive peptides in insect protein may support gut health and enhance immune function in pets. Chitin and other fiber components act as prebiotics, fostering beneficial gut microbiota, and can serve as functional ingredients and natural antioxidants in pet food. Hence, alternative proteins offer sustainability, enhanced nutrition, and versatile functional ingredients, and act as natural antioxidants, enabling their use in hybrid pet-food formulations.

**Table 4:** Patents relating to alternative proteins for pet foods.

Protein Source	Food Process	Product or Target Application	Major Claim	Patent Types	Country	Patent Name	Ref
Pea protein, Animal muscle-derived protein, Egg protein	• Craped surface heat exchangers • Steam tunnel cooking	• Wet pet food • Dry pet food	• Meat-analogue pet-food chunk development • Combined animal, egg, and pea protein source • Multi-scraped heat exchanger meat chunk production • Pet food with visible botanical inclusions (wet/dry)	Invention Patent	United States (US)	Meat-like Pet-food Chunks	[57]
Soy protein, Wheat gluten, Corn gluten, Fish meat, Livestock, Meat, Dairy proteins, Egg proteins	• Extrusion • Heat Processing • High-pressure kneading	• Wet pet food	• Hybrid meat-analogue pet food (flakes) • Regulating methionine in cat food for optimal nutrition • Structured plant-protein meat analogues • Extrusion for efficient meat analogues	Invention Patent	Japan (JP)	Pet Food	[58]
Soybeans, Wheat, Rice, Barley, Rye, Red beans, Chicken, Beef, Duck, Pork, Anchovy, Pollock, Mackerel	• Extrusion • Baking	• Wet pet food • Dry pet food	• Achyranthes Japonica extract for joint health and antioxidative benefits • Herbal palatability enhancement. • Nutritionally complete formulation	Invention Patent	South Korea (KR)	Pet Feed Composition Comprising Achyranthes Japonica Nakai Extract	[59]
Textured soy protein, Pea, Wheat, King oyster, Mushroom, Egg yolk, Animal by-products	Extrusion	Pet snacks	• High-protein, plant-based meat-replacement pet snacks • Soy, wheat, pea protein; amino acid balance • Natural flavor enhancement for palatability (seaweed, rosemary oil, animal-derived palatability agents) • Pet-health functional ingredient incorporation	Invention Patent	China (CN)	High-Protein Dog Snack, Cat Snack, and Preparation Method Thereof	[60]
Pea protein, Low-fat yogurt, Powdered chicken breast, Lean beef, Salmon, Bass, Crayfish	Microencapsulation technology	Functional pet food	• Microencapsulated bioactives for sustained health benefits • Functional ingredient inclusion for anti-inflammatory, antioxidative, and metabolic effects • Balanced macronutrients with ≥55% protein for pet health	Invention Patent	China (CN)	Formula and Preparation Method of Pet Food with Blood Sugar and Blood Fat Reducing Function	Shanghai Liangrun [61]
Fish, Aquatic mammals, Crustaceans, Mollusks	Enzymatic hydrolysis	Ingredient	• Enzymatic hydrolysis of aquatic proteins with natural antioxidant fortification • Enhanced oxidative stability and bioactive potential • Reduced APH bitterness enhances commercial viability	Invention Patent	United States (US)	Use of natural antioxidants during enzymatic hydrolysis of aquatic protein to obtain high quality aquatic protein hydrolysates	[62]
Single-cell protein, Hydrolyzed protein, Protein concentrates, Peptides	Microbial fermentation Protein extracts, hydrolysates, and peptides	Meat alternatives	• Hydrolyzed and enriched microbial protein applications	Invention Patent	South Korea (KR)	High Protein Food Composition	[63]
Pea protein, Soy protein, Wheat gluten, Chicken, Beef, Fish protein, Meat by-products	Extrusion	Ingredient	• Innovative protein blend for pet-food development • Advanced processing for nutrient bioavailability	Invention Patent	United States (US)	Pet-Food Product	[64]
Salmon, Sardines, Mussels, Chicken, Duck	• Cooking process • Vacuum packaging	Ingredient	• Enhanced protein, natural nutrition for pet wellness • Cold chain storage	Invention Patent	China (CN)	Formula and Preparation Method of Fresh Meat Food for Pets	[65]
Chlorella, DHA, EPA, Aloe, Spirulina, Bamboo leaf, Green tea extract, Licorice root, Mulberry leaf, Meat powder	Extrusion	• Ingredient	• Chlorella incorporation for enhanced protein quality and bioavailability • Improved health, digestion, and overall vitality compared to conventional dog food. • Antioxidant and metabolic botanicals	Invention Patent	South Korea (KR)	Feed of Dog with Chlorella and Manufacturing Method Thereof	[66]
Microalgae (Porphyridium cruentum), Animal blood plasma	Extrusion	• Wet pet food	• Microalgae-enhanced blood plasma binder for pet food	Invention Patent	Europe	Pet-Food Product Comprising Microalgae as Binder	[67]
Water-soluble insect protein hydrolysate	Hydrolysis	• Pet food • Livestock feed • Aquaculture feed	• Enhanced animal feed via water-soluble insect protein hydrolysate	Invention Patent	Netherlands (NL)	Hydrolysate of Water-Soluble Insect Proteins	[68]

## 6. Challenges and Future Opportunities

The integration of plant, aquatic, insect, and cell-based sources into pet food presents a compelling pathway towards a more sustainable future for the industry. The opportunity lies in mitigating the significant environmental impact of traditional livestock farming, offering novel ingredients for pets with sensitivities, and potentially creating more resource-efficient and ethically sound food sources. These alternatives can tap into a growing consumer demand for sustainable and health-conscious pet-food options, fostering innovation and differentiation in the market. Furthermore, they offer a buffer against supply chain vulnerabilities associated with conventional animal agriculture. However, significant challenges remain. Ensuring sufficient nutritional adequacy and bioavailability of these novel proteins to meet the specific needs of different pet species and life-stages is paramount and requires rigorous scientific investigation and formulation. Consumer acceptance, influenced by perceptions of novelty, taste, and safety, needs to be carefully cultivated through transparent communication and education about the benefits. In addition to sensory factors, many pet owners evaluate pet foods based on psychological and ethical considerations such as perceived naturalness, ingredient transparency, environmental sustainability, and animal welfare. While some consumers are willing to pay a premium for pet foods that support sustainability or animal welfare, others remain hesitant due to unfamiliarity or perceived risks associated with novel ingredients. It is important to build trust through clear labeling, straightforward communication, independent certifications, and educational efforts that highlight the benefits and safety of alternative protein sources. Although some alternative proteins may reduce the palatability of pet food, such as the distinctive taste or salty and bitter notes from algae-based proteins [10], or the lack of key flavor compounds in mealworm protein [51], these issues can potentially be addressed using palatants. Scaling up production to meet industry demands while maintaining cost-effectiveness and quality control presents a considerable hurdle. Finally, the regulatory landscape for these alternative protein sources is still evolving and lacks global harmonization, potentially hindering market access and innovation. Insect-based proteins are authorized for use in pet food in the European Union under specific conditions through the Novel Food framework (Regulation (EU) 2015/2283), which mandates pre-market approval for species such as yellow mealworm and house cricket following safety evaluations by EFSA. In contrast, the US currently lacks a harmonized federal policy for insect proteins in pet food. Instead, the use of insects is subject to Generally Recognized as Safe (GRAS) notifications and varies by state, creating uncertainty for manufacturers [69]. In Asia, Thailand permits the use of insects in both human food and pet feed, having established frameworks for insect farming and processing. In contrast, Japan allows edible insects for human consumption but currently lacks explicit regulations for their use in pet food, creating uncertainty relative to their formal adoption in the companion-animal sector [70]. When formulating insect-based pet food, it is crucial to consider potential challenges like allergenicity and the accumulation of toxins in insects. Insects belong to the phylum Arthropoda, which also includes common allergens like dust mites and crustaceans (e.g., shrimp, crabs) [71]. This shared protein means that pets allergic to dust mites or crustaceans could exhibit allergic reactions to insect-based pet food due to shared allergenic epitopes. Research, for instance, shows that IgEs from canine sera can bind to mealworm proteins, indicating a possibility of cross-reactivity in mite-allergic dogs [72]. Therefore, while insects offer a novel protein source, insect-based diets may not be truly hypoallergenic for all animals, especially those with pre-existing arthropod allergies. Furthermore, insects can bioaccumulate heavy metals like cadmium (Cd), lead (Pb), arsenic (As), and mercury (Hg) from their feed. The extent of this accumulation varies depending on the insect species, the composition of their substrate, and environmental factors. For example, studies have demonstrated significant accumulations of cadmium and lead in various insect species, underscoring the critical need for strict control over the insect feed material to maintain contaminant levels below the safety thresholds for animal feed [73]. These potential safety concerns must be carefully addressed in pet-food production.

In the US, cultured meat is regulated under the joint oversight of the FDA and USDA-FSIS, but clear guidance regarding its use in pet food remains limited. Some pet-food manufacturers have begun exploring in vitro culture methods as alternative protein sources. On 10 July 2024, the UK had the world’s first regulatory approval for a cultivated meat product intended to be sold as a pet food, developed by the company Meatly [74]. The cell-cultured meat industry faces a long journey toward achieving profitable large-scale operations. The three primary production costs are cell culture media, bioreactors, and processing equipment [75]. Moreover, consumers for whom the price of cultivated pet food was the primary concern were significantly more inclined to feed it to their pets [54].

The successful transition towards alternative proteins in pet food hinges on collaborative efforts between researchers, manufacturers, regulatory bodies, and consumers. Addressing the nutritional, technological, and perceptual challenges while capitalizing on the sustainability-related and ethical opportunities will pave the way for a pet-food industry that is not only nourishing for our companions but also responsible towards the planet. Further research is also crucial to understand the long-term health impacts of these proteins on pets to ensure safety and efficacy.

## 7. Conclusions

The exploration of alternative proteins for pet food presents a promising avenue for addressing sustainability challenges while maintaining high nutritional standards for pets. Plant-based, insect-derived, aquatic-source-derived, and cell-based proteins can serve as sustainable alternatives to traditional animal proteins. Each alternative protein source presents unique benefits, such as improved digestibility, enhanced gut health, and the provision of essential amino acids and bioactive compounds. However, factors including palatability, regulatory approval, production scalability, and consumer acceptance remain critical considerations for their widespread adoption. Future research should focus on optimizing the processing techniques in order to enhance the functionality and bioavailability of alternative proteins in pet-food formulations. Additionally, further studies are needed to assess the long-term health impacts of these proteins on pets to ensure safety and efficacy. Collaboration between researchers, pet-food manufacturers, and regulatory bodies will be essential in driving innovation and acceptance in this evolving field. Overall, the integration of alternative proteins into pet-food products has the potential to revolutionize the industry by offering sustainable and nutritionally balanced options. As technological advancements continue to improve ingredient functionality and consumer perception evolves, alternative proteins are likely to become an integral component of the future pet-food market.

## Figures and Tables

**Table 1:** Plant-based proteins in pet foods.

Protein Source	Food Process	Product	Factors of Investigation	Method of Investigation	Major Finding	Ref.
Soy protein isolate and wheat gluten (MaSoy), pea protein isolates and wheat gluten (MaPea).	Sheared (120 °C, 30 min, 30 rpm) and sterilized (125.5 °C, 1.36 bar, 26 min)	Canned chunk meat analogue	• Protein source	• Amino acid content	• Amino acids based on nitrogen decreased on average 31.0 ± 1.86% for MaSoy and 14.4 ± 2.76% for MaPea. • Plant-based protein quality stable after heating.	[29]
Soy protein isolate and wheat gluten.	Sheared (120 °C, 30 min, 30 rpm), sterilized (124.5 °C, 1.28 bar, 36.6 min), and stored (21 °C, 7 or 28 days)	Couette-cell meat analogue (CCMA)	• Protein source	• Proximate analysis • Texture profile analysis	• Fibrous texture retained post-sterilization. • Sterilized meat analogue texture equivalent to canned pet food.	[28]
Wheat gluten and soy protein isolate (MaSoy), wheat gluten and pea protein isolate (MaPea), and wheat gluten and faba bean concentrate (MaFaba).	Sheared (120 °C, 30 min) and sterilized (126.6 °C, 21 min, 1.36 bar)	Canned chunk meat analogue in water and gravy	• Protein source	• In vitro protein digestion • In vitro dry matter and nitrogen digestibility	• Plant-based products exhibited nutrient digestibility and phosphorus solubility comparable to or exceeding the reference pet food. • Dry matter crude protein: MaFaba (72.8%), MaPea (intermediate), MaSoy (87.8%). • Meat analogue crude fat: Low (max. 7.6% DM, MaPea gravy).	[30]

## Data Availability

The original contributions presented in the study are included in the article/supplementary material, further inquiries can be directed to the corresponding author.

## References

[1] Tiffany S., Parr J.M., Templeman J., Shoveller A.K., Manjos R., Yu A., Verbrugghe A. (2019). Assessment of dog owners’ knowledge relating to the diagnosis and treatment of canine food allergies. Can. Vet. J..

[2] Biel W., Natonek-Wisniewska M., Kepinska-Pacelik J., Kazimierska K., Czerniawska-Piatkowska E., Krzyscin P. (2022). Detection of chicken DNA in commercial dog foods. BMC Vet. Res..

[3] Robinson G.H.J., Balk J., Domoney C. (2019). Improving pulse crops as a source of protein, starch and micronutrients. Nutr. Bull..

[4] Pali-Scholl I., De Lucia M., Jackson H., Janda J., Mueller R.S., Jensen-Jarolim E. (2017). Comparing immediate-type food allergy in humans and companion animals-revealing unmet needs. Allergy.

[5] Mueller R.S., Olivry T., Prelaud P. (2016). Critically appraised topic on adverse food reactions of companion animals (2): Common food allergen sources in dogs and cats. BMC Vet. Res..

[6] Durdakova M., Kolackova M., Ridoskova A., Cernei N., Pavelicova K., Urbis P., Richtera L., Pelcova P., Adam V., Huska D. (2024). Exploring the potential nutritional benefits of *Arthrospira maxima* and *Chlorella vulgaris*: A focus on vitamin B_12_, amino acids, and micronutrients. Food Chem..

[7] Makkar H.P.S., Tran G., Heuzé V., Ankers P. (2014). State-of-the-art on use of insects as animal feed. Anim. Feed Sci. Technol..

[8] Govorushko S. (2019). Global status of insects as food and feed source: A review. Trends Food Sci. Technol..

[9] Kara K., Kahraman O., İnal F., İnanç Z.S., Yilmaz Öztaş S., Alataş M.S., Ahmed I. (2025). Digestion, faeces microbiome, and selected blood parameters in dogs fed extruded food containing Black soldier fly (*Hermetia illucens*) meal. Ital. J. Anim. Sci..

[10] Mota C.S., Cabrita A.R., Yergaliyev T., Camarinha-Silva A., Almeida A., Abreu H., Silva J., Fonseca A.J., Maia M.R.G. (2024). Macroalgae and microalga blend in dogs’ food: Effects on palatability, digestibility, and fecal metabolites and microbiota. Algal Res..

[11] Matassa S., Boon N., Pikaar I., Verstraete W. (2016). Microbial protein: Future sustainable food supply route with low environmental footprint. Microb. Biotechnol..

[12] Gahukar R.T. (2016). Edible Insects Farming: Efficiency and Impact on Family Livelihood, Food Security, and Environment Compared With Livestock and Crops. Insects as Sustainable Food Ingredients.

[13] Failla M., Hopfer H., Wee J. (2023). Evaluation of public submissions to the USDA for labeling of cell-cultured meat in the United States. Front. Nutr..

[14] Webb D., Dogan H., Li Y., Alavi S. (2023). Physico-Chemical Properties and Texturization of Pea, Wheat and Soy Proteins Using Extrusion and Their Application in Plant-Based Meat. Foods.

[15] Lidzba N., Garcia Arteaga V., Schiermeyer A., Havenith H., Muranyi I., Schillberg S., Lehmann J., Ueberham E. (2021). Development of Monoclonal Antibodies against Pea Globulins for Multiplex Assays Targeting Legume Proteins. J. Agric. Food Chem..

[16] Yang J., Zamani S., Liang L., Chen L. (2021). Extraction methods significantly impact pea protein composition, structure and gelling properties. Food Hydrocoll..

[17] Miranda C.G., Rodrigues R.M., Pereira R.N., Speranza P., Kurozawa L.E., Vicente A.A., Sato A.C.K. (2023). Influence of ohmic heating on lentil protein structure and protein-pectin interactions. Innov. Food Sci. Emerg. Technol..

[18] Johansson M., Johansson D., Ström A., Rydén J., Nilsson K., Karlsson J., Moriana R., Langton M. (2022). Effect of starch and fibre on faba bean protein gel characteristics. Food Hydrocoll..

[19] Nagarajan D., Varjani S., Lee D.-J., Chang J.-S. (2021). Sustainable aquaculture and animal feed from microalgae—Nutritive value and techno-functional components. Renew. Sustain. Energy Rev..

[20] Sharma B., Yadav D.K., Malakar S., Singh S., Sharma M., Suri S., Sridhar K. (2024). Insect proteins—Production technologies, bio-functional, and food applications: A perspective. Food Biosci..

[21] Akhtar Y., Isman M.B. (2018). Insects as an Alternative Protein Source. Proteins in Food Processing.

[22] Kipkoech C. (2023). Beyond proteins—Edible insects as a source of dietary fiber. Polysaccharides.

[23] Jacuńska W., Biel W., Zych K. (2024). Evaluation of the Nutritional Value of Insect-Based Complete Pet Foods. Appl. Sci..

[24] Acuff H.L., Dainton A.N., Dhakal J., Kiprotich S., Aldrich G. (2021). Sustainability and Pet Food: Is There a Role for Veterinarians?. Vet. Clin. N. Am. Small Anim. Pract..

[25] Knight A. (2023). The relative benefits for environmental sustainability of vegan diets for dogs, cats and people. PLoS ONE.

[26] Krintiras G.A., Göbel J., Jan van der Goot A., Stefanidis G.D. (2015). Stefanidis. Production of structured soy-based meat analogues using simple shear and heat in a Couette Cell. J. Food Eng..

[27] Sha L., Xiong Y.L. (2020). Plant protein-based alternatives of reconstructed meat: Science, technology, and challenges. Trends Food Sci. Technol..

[28] Wehrmaker A.M., Bosch G., van der Goot A.J. (2021). Effect of sterilization and storage on a model meat analogue pet food. Anim. Feed Sci. Technol..

[29] Wehrmaker A.M., Zenker H.E., de Groot W., Sanders M., van der Goot A.J., Janssen A.E.M., Keppler J., Bosch G. (2022). Amino Acid Modifications During the Production (Shearing, Sterilization) of Plant-Based Meat Analogues: An Explorative Study Using Pet Food Production as an Example. ACS Food Sci. Technol..

[30] Wehrmaker A.M., de Groot W., Jan van der Goot A., Keppler J.K., Bosch G. (2024). In vitro digestibility and solubility of phosphorus of three plant-based meat analogues. J. Anim. Physiol. Anim. Nutr..

[31] Cabrita A.R., Guilherme-Fernandes J., Spínola M., Maia M.R., Yergaliyev T., Camarinha-Silva A., Fonseca A.J. (2023). Effects of microalgae as dietary supplement on palatability, digestibility, fecal metabolites, and microbiota in healthy dogs. Front. Vet. Sci..

[32] Dalmonte T., Vecchiato C.G., Biagi G., Fabbri M., Andreani G., Isani G. (2023). Iron Bioaccessibility and Speciation in Microalgae Used as a Dog Nutrition Supplement. Vet. Sci..

[33] Stefanutti D., Tonin G., Morelli G., Zampieri R.M., La Rocca N., Ricci R. (2023). Oral palatability and owners’ perception of the effect of increasing amounts of Spirulina (*Arthrospira platensis*) in the diet of a cohort of healthy dogs and cats. Animals.

[34] Satyaraj E., Reynolds A., Engler R., Labuda J., Sun P. (2021). Supplementation of diets with spirulina influences immune and gut function in dogs. Front. Nutr..

[35] Srinivas K.Y., Das A., Reddy P.B., Verma A.K. (2024). Supplementation of tropical red seaweeds improved gut health indices, antioxidant status and immunity in adult dogs. J. Appl. Phycol..

[36] Isidori M., Rueca F., Massacci F.R., Diaferia M., Giontella A., Caldin M., Furlanello T., Corbee R.J., Mannucci G., Pezzotti G. (2021). The use of Ascophyllum nodosum and Bacillus subtilis C-3102 in the management of canine chronic inflammatory enteropathy. A Pilot study. Anim..

[37] Gawor J.P., Wilczak J., Svensson U.K., Jank M. (2021). Influence of Dietary Supplementation with a Powder Containing AN ProDen™ (*Ascophyllum nodosum*) Algae on Dog Saliva Metabolome. Front. Vet. Sci..

[38] Mohamad Yusof L., Ahmad H., Hassim H.A., Mustaffa-Kamal F., Omar S., Zainundin N.K., Padam B.S. (2024). A study on the impact of diet supplementation of fermented dried seaweed powder (*Kappaphycus alvarezii*) on healthy cat gut performance, skin and hair coat conditions, and behaviour. Vet. Res. Commun..

[39] Cabrita A.R., Guilherme-Fernandes J., Valente I.M., Almeida A., Lima S.A., Fonseca A.J., Maia M.R. (2022). Nutritional composition and untargeted metabolomics reveal the potential of *Tetradesmus obliquus*, *Chlorella vulgaris* and *Nannochloropsis oceanica* as valuable nutrient sources for dogs. Animals.

[40] Pinna C., Vecchiato C.G., Grandi M., Stefanelli C., Zannoni A., Biagi G. (2021). Seaweed supplementation failed to affect fecal microbiota and metabolome as well as fecal IgA and apparent nutrient digestibility in adult dogs. Animals.

[41] Isidori M., Rueca F., Trabalza-Marinucci M. (2019). Palatability of extruded dog diets supplemented with *Ascophyllum nodosum* L. (Fucaceae, Phaeophyceae). J. Appl. Phycol..

[42] Zhang M., Mo R., Li M., Qu Y., Wang H., Liu T., Liu P., Wu Y. (2023). Comparison of the effects of enzymolysis seaweed powder and *Saccharomyces boulardii* on intestinal health and microbiota composition in kittens. Metabolites.

[43] Bosch G., Loureiro B., Schokker D., Kar S., Paul A., Sluczanowski N. (2024). Black soldier fly larvae meal in an extruded food: Effects on nutritional quality and health parameters in healthy adult cats. J. Insects Food Feed.

[44] Zhang Q., Sun H., Gao Z., Zhao H., Peng Z., Zhang T. (2024). Evaluation of Effective Energy Values of Six Protein Ingredients Fed to Beagles and Predictive Energy Equations for Protein Feedstuff. Animals.

[45] Areerat S., Chundang P., Lekcharoensuk C., Patumcharoenpol P., Kovitvadhi A. (2023). Insect-based diets (house crickets and mulberry silkworm pupae): A comparison of their effects on canine gut microbiota. Vet. World.

[46] Penazzi L., Schiavone A., Russo N., Nery J., Valle E., Madrid J., Martinez S., Hernandez F., Pagani E., Ala U. (2021). In vivo and in vitro digestibility of an extruded complete dog food containing black soldier fly (*Hermetia illucens*) larvae meal as protein source. Front. Vet. Sci..

[47] Mouithys-Mickalad A., Schmitt E., Dalim M., Franck T., Tome N.M., van Spankeren M., Serteyn D., Paul A. (2020). Black soldier fly (*Hermetia illucens*) larvae protein derivatives: Potential to promote animal health. Animals.

[48] Seo K., Cho H.-W., Chun J., Jeon J., Kim C., Kim M., Park K., Kim K. (2021). Evaluation of fermented oat and black soldier fly larva as food ingredients in senior dog diets. Animals.

[49] Jian S., Zhang L., Ding N., Yang K., Xin Z., Hu M., Zhou Z., Zhao Z., Deng B., Deng J. (2022). Effects of black soldier fly larvae as protein or fat sources on apparent nutrient digestibility, fecal microbiota, and metabolic profiles in beagle dogs. Front. Microbiol..

[50] Abd El-Wahab A., Meyer L., Kölln M., Chuppava B., Wilke V., Visscher C., Kamphues J. (2021). Insect larvae meal (*Hermetia illucens*) as a sustainable protein source of canine food and its impacts on nutrient digestibility and fecal quality. Animals.

[51] Feng T., Hu Z., Tong Y., Yao L., Zhuang H., Zhu X., Song S., Lu J. (2020). Preparation and evaluation of mushroom (*Lentinus edodes*) and mealworm (*Tenebrio molitor*) as dog food attractant. Heliyon.

[52] Bosch G., Vervoort J., Hendriks W. (2016). In vitro digestibility and fermentability of selected insects for dog foods. Anim. Feed Sci. Technol..

[53] Stefanutti D. (2024). A new protein source for pet food: Cultivated meat. Companion Anim..

[54] Oven A., Yoxon B., Milburn J. (2022). Investigating the market for cultivated meat as pet food: A survey analysis. PLoS ONE.

[55] Andy C. (2023). Eat Just Eyes Cultivated-Meat Scale-Up After New Singapore Nod, in Just Food.

[56] Zafalon R.V.A., Risolia L.W., Vendramini T.H.A., Rodrigues R.B.A., Pedrinelli V., Teixeira F.A., Brunetto M.A. (2020). Nutritional inadequacies in commercial vegan foods for dogs and cats. PLoS ONE.

[57] Ray T.K. (2020). Meat-like pet food chunks.

[58] Miyamoto K. Pet food.

[59] Hwang K.T. (2023). Pet feed composition comprising Achyranthes japonica Nakai extract.

[60] Qingda Yalute Food Co., Ltd. (2022). High-Protein Dog Snack, Cat Snack, and Preparation Method Thereof.

[61] Gao H. (2024). Formula and preparation method of pet food with blood sugar and blood fat reducing function.

[62] Halldorsdottir S.M., Kristinsson H.G., Jonsdottir R. (2015). Use of natural antioxidants during enzymatic hydrolysis of aquatic protein to obtain high-quality aquatic protein hydrolysates.

[63] (2022). Air Protein Inc. High-protein food composition.

[64] Mars Inc. (2024). Pet food product.

[65] Shanghai Peitong Technology Co., Ltd. (2023). Formula and preparation method of fresh meat food for pets.

[66] (2008). Daesang Farmsco Co., Ltd., Feed of dog with Chlorella and manufacturing method thereof.

[67] Mars, Incorporated (2024). Pet food product comprising microalgae as binder.

[68] Netherlands Patent Office (2022). Hydrolysate of water-soluble insect proteins.

[69] Precup G., Ververis E., Azzollini D., Rivero-Pino F., Zakidou P., Germini A. (2022). The safety assessment of insects and products thereof as novel foods in the European Union. Novel Foods and Edible Insects in the European Union.

[70] Lee J.H., Kim T.K., Cha J.Y., Jang H.W., Yong H.I., Choi Y.S. (2022). How to develop strategies to use insects as animal feed: Digestibility, functionality, safety, and regulation. J. Anim. Sci. Technol..

[71] Ni D. (2025). Allergenic potential of insect proteins: Cross-reactivity, detection, degradation, and implications for food safety and labelling. Food Sci. Anim. Prod..

[72] Premrov Bajuk B., Zrimšek P., Kotnik T., Leonardi A., Križaj I., Jakovac Strajn B. (2021). Insect protein-based diet as potential risk of allergy in dogs. Animals.

[73] Van der Fels-Klerx H.J., Camenzuli L., Belluco S., Meijer N., Ricci A. (2018). Food safety issues related to uses of insects for feeds and foods. Compr. Rev. Food Sci. Food Saf..

[74] Johnson H., Monaco A. (2025). Global developments in the regulation of cultivated meat: A comparative study of the EU, Singapore, US and Australia and New Zealand. Rev. Eur. Comp. Int. Environ. Law..

[75] Garrison G.L., Biermacher J.T., Brorsen B.W. (2022). How much will large-scale production of cell-cultured meat cost?. J. Agric. Food Res..

